# Base Plate Preheating Effect on Microstructure of 316L Stainless Steel Single Track Deposition by Directed Energy Deposition

**DOI:** 10.3390/ma14185129

**Published:** 2021-09-07

**Authors:** Abhilash Kiran, Martina Koukolíková, Jaroslav Vavřík, Miroslav Urbánek, Jan Džugan

**Affiliations:** COMTES FHT a.s., Průmyslová 995, 33441 Dobřany, Czech Republic; martina.koukolikova@comtesfht.cz (M.K.); jaroslav.vavrik@comtesfht.cz (J.V.); miroslav.urbanek@comtesfht.cz (M.U.); jan.dzugan@comtesfht.cz (J.D.)

**Keywords:** directed energy deposition, 316L stainless steel, additive manufacturing, microstructures

## Abstract

The microstructural morphology in additive manufacturing (AM) has a significant influence on the building structure. High-energy concentric heat source scanning leads to rapid heating and cooling during material deposition. This results in a unique microstructure. The size and morphology of the microstructure have a strong directionality, which depends on laser power, scanning rate, melt pool fluid dynamics, and material thermal properties, etc. The grain structure significantly affects its resistance to solidification cracking and mechanical properties. Microstructure control is challenging for AM considering multiple process parameters. A preheating base plate has a significant influence on residual stress, defect-free AM structure, and it also minimizes thermal mismatch during the deposition. In the present work, a simple single track deposition experiment was designed to analyze base plate preheating on microstructure. The microstructural evolution at different preheating temperatures was studied in detail, keeping process parameters constant. The base plate was heated uniformly from an external heating source and set the stable desired temperature on the surface of the base plate before deposition. A single track was deposited on the base plate at room temperature and preheating temperatures of 200 °C, 300 °C, 400 °C, and 500 °C. Subsequently, the resulting microstructural morphologies were analyzed and compared. The microstructure was evaluated using electron backscattered diffraction (EBSD) imaging in the transverse and longitudinal sections. An increase in grain size area fraction was observed as the preheating temperature increased. Base plate preheating did not show influence on grain boundary misorientation. An increase in the deposition depth was noticed for higher base plate preheating temperatures. The results were convincing that grain morphology and columnar grain orientation can be tailored by base plate preheating.

## 1. Introduction

Directed energy deposition (DED) is a well-known additive manufacturing (AM) process because of its unique application. The material in the form of powder/wire is supplied directly to the melt pool created by a high-energy laser/electron beam on the target surface [1,2,3]. This enables the depositing of the material on an irregular surface. This technique allows a combination of materials to be supplied into the melt pool. Therefore, this technique has applications like repairing engineering components, surface coating, and compositionally graded materials deposition. These applications attract vital interest to the microstructure development on a deposited material and also on the base plate. Microstructural tuning in order to tailor mechanical properties has great importance. It is evident from the extensive literature in metal AM [4,5,6,7,8,9,10,11,12,13,14]. Studies on grain morphology, melt pool solidification, solidification texture, the temperature gradient in the melt pool, the affect of cooling rate on morphology, and size of the microstructure are reported for AM [15,16,17,18].

The final microstructure of the deposited structure has an influence on the tensile strength and ductility. Higher tensile strength and lower ductility for DED processed structures appear due to a finer microstructure [19]. T. Wang et al. reported heterogeneous nucleation in the melt pool [11]. Two dominated microstructures were generated in the melt pool solidification. Epitaxial growth of equiaxed and columnar grain was noticed at the upper surface and melt pool bottom, respectively, for the single track deposition in the transverse view. Base plate grains were influenced by the hot melt pool. Grains were restructured due to rapid heating and cooling from a large temperature gradient. In the multilayer deposition process, each beneath layer acted as a heat affected zone (HAZ). A solidified layer was partially or completely remelted, which led to recrystallization, and this had an influence on the microstructure and mechanical properties of the deposited structure. Columnar grain size and orientation were the predominant factors in solid structure mechanical properties. The base plate preheating had influence on the HAZ. It was reported that the depth of the HAZ increased by an average of 400 μm for a thinner base plate [19]. Repairing and surface coating application has attracted interest in the deposited and base plate grain structure [20]. Research on the surface coating application of DED has gained interest [21,22]. Controlling the HAZ depth in DED can extend these applications, including thin substrate coating. It is important to take into account the microstructural modification on the repairing component and in the surface coating applications.

Grain size and morphology in the melt pool are predominantly influenced by the temperature gradient. A grain morphology is determined by two parameters: temperature gradient within the liquid phase (G) and the velocity of the solidification front (R). Depending on the desired microstructure, G and R values can be controlled mainly by process parameters such as laser power and scanning velocity [5]. The product of G*R determines the cooling rate in the solidification interval. A higher cooling rate tends to the development of fine columnar structure and directionally oriented grain growth, whereas the ratio of G and R determines the morphology of the grains. A higher G/R ratio is desired to achieve ordered columnar grains, which can be reached by cooling the base plate. The cooling of the base plate during deposition contributes to altering heat dissipation from the melt pool, which changes solidifying crystal orientation. This method results in lowered solidification cracking, whereas thermal stress is inevitable due to the higher cooling rate [23]. On the other hand, a lower G/R ratio is desired to achieve columnar to equiaxed transition (CET) by homogeneous grain nucleation in the melt pool. One of the techniques to achieve a lower G/R ratio is preheating the base plate. Varying process parameters to tune the microstructure alter melt pool dimensions. Grain morphology can be controlled by preheating without changing process parameters. Preheating the base plate reduces larger thermal gradients at the melt pool to the base plate/beneath layer intersection.

The cooling rate (G*R) while depositing a 3D solid structure varies over building direction [14]. A higher cooling rate was evident in the layers near the base plate. It was due to deposition on the base plate, which was at room temperature and acted as a faster heat sink. This might lead to different grain structures in the AM solid components. Variation of microstructure along the build direction changes the microhardness. The microhardness is higher near the base plate layer and at the top. This contributes to the fine microstructure created due to the large thermal gradient. Heat buildup in the deposited structure occurs due to continuous deposition [24]. Therefore, a higher temperature could appear during the middle section deposition. This results in the coarse grains in the middle of the structure, which yield relatively low microhardness [25]. An ideal preheating temperature for the base plate could help to develop a homogeneous microstructure in the deposited structure.

High preheating temperature appeared to be more effective for DED compared to selective laser melting (SLM) [26,27,28]. Corbin et al. studied the influence of Ti-6AL-4V substrate thickness on part distortion at 300 °C preheating and reported that preheating on the thin base plate was more effective [29]. Lu et al. studied different scanning strategies for preheating to reduce residual stress and distortion. At 700 °C, they reported that there was lower initial distortion and residual tensile stress near the base plate. The deposition at 700 °C base plate preheating and 500 °C ambient temperature resulted in the final deformation and residual stress mitigation by 90.1% and 80.2%, respectively [30]. Though the preheating effect on residual stress and distortion were studied in detail by several authors, a preheating effect on microstructure has not been reported to the same extent.

In the presented paper, a single track of 316L stainless steel powder was deposited on the base plate made of the same material. Subsequently, the microstructural morphology and melt pool dimensions were analyzed for different base plate preheating temperatures. The process parameters for all cases were kept constant to evaluate solely the influence of the base plate preheating on the microstructural evolution. EBSD grain mapping for a single track in transversal and longitudinal directions was investigated. A grain shape and size dependence on the base plate preheating were studied. The fusion zone (FZ) regions were evaluated. The key important factors for AM like grain size, FZ dimensions, and grain orientation for all cases were evaluated.

## 2. Experimental Procedure

### 2.1. Experimental Setup

**Figure 1:**
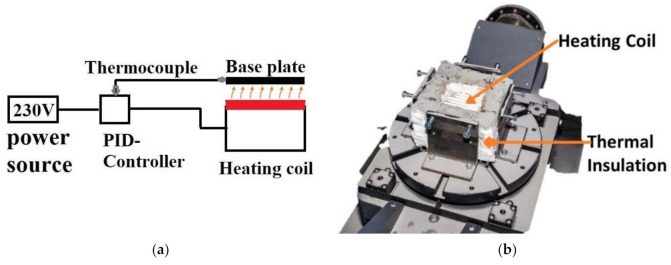
(**a**) Schematic diagram of heating system (**b**) Preheating setup.

An external preheating setup was designed for the InssTek MX-600 (InssTek, Daejeon, Korea) metallic deposition system, which is equipped with a DED process technology. A base plate holder setup was modified from standard equipment available for this machine. The heating element (Elstein, Northeim, Germany) was placed on the base plate holder using a steel frame for safety reasons. It has an operating temperature of up to 900 °C and 1000 W of maximum power. A block diagram of the heating element setup is depicted in Figure 1a. Fiberglass insulation was built to the height of 45 mm from the surface of the heating element as shown in Figure 1b. The base plate was placed firmly with the use of bolts. The base plate surface orientation was maintained horizontal to the height of the fiberglass insulation. The four-frame structure was designed to provide rigid support for the base plate. The frames were bolted to the base plate holder of the machine. This arrangement created a direct passage between the heating element and bottom face of the base plate surrounded by fiberglass insulation of thickness 40 mm. The insulation helped to avoid heat loss and maintain the constant desired temperature of the base plate during the deposition.

**Figure 2:**
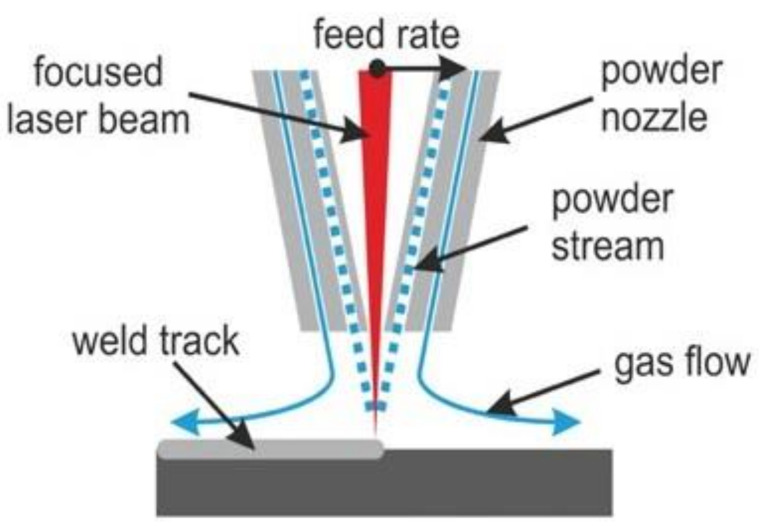
Schematic diagram of DED process. Reprinted from Ref. [31].

**Figure 3:**
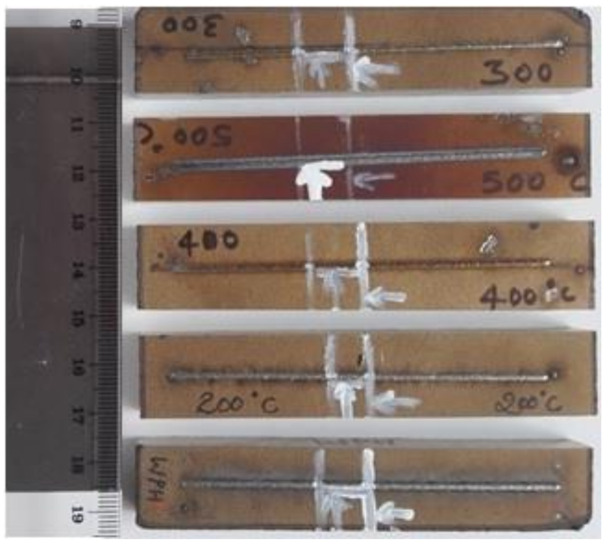
Single-track deposition on deferent base plate preheating temperature.

InssTek MX-600 utilizes powder as the feedstock material. A laser head has multiple concentric cones, which provide passage for powder and shield gas flow. The powder is directed to the laser point contact with the base plate, where it generates the melt pool. Shielding gas at the rate of 5 L/min was fed around the laser and powder path to separate the melt pool from the external environment to avoid oxidation. The schematic diagram of the DED working principle is illustrated in Figure 2. The melt pool is exposed to ambient temperature when the laser head moves with a defined feed rate and direction. The deposited material solidifies and forms a track as shown in Figure 3.

A base plate of dimensions 100 mm × 100 mm × 10 mm was heat-treated and cut into 5 pieces. This was to maintain the history of manufacturing since grain morphology in the base plate should be the same for all cases. The base plates were heated from room temperature to respective cases independently before deposition. A single-track length of 80 mm was deposited on the base plate of dimensions 100 mm × 20 mm × 10 mm. The single track was cut in the middle along the deposition direction for transverse section examination as shown in Figure 3. Two sets for each preheating temperature case were deposited for repeatability and to evaluate the precise control on preheating conditions. One set was considered for detail investigation. The material was deposited for five different cases, namely the base plate at room temperature and preheated temperatures of 200 °C, 300 °C, 400 °C, and 500 °C. The material was deposited after the base plate surface temperature was stable in respective cases. The temperature of the base plate surface was measured using a thermocouple type “K” [24]. The rate of temperature increase for preheating the base plate and cooling rate after deposition was monitored using real-time thermocouple data.

The DED process parameters are listed in Table 1 for all five cases. Below process parameters were provided from DED machine producer.

### 2.2. Microstructural Evaluation

A conventional metallographic preparation involved grinding and subsequent polishing by a standard procedure performed on a Tegramin 30 (Struers GmbH, Ballerup, Denmark). The microstructures were revealed by etching in V2A solution and photographed using a light microscope Nikon Eclipse MA200 (Nikon, Tokyo, Japan) equipped with an NIS Elements 5.2 digital image processing and analysis software (Nikon, Tokyo, Japan). Detailed microstructural observation and electron backscatter diffraction (EBSD) was performed on a scanning electron microscope JEOL IT 500 HR (JEOL Ltd., Tokyo, Japan) with EDAX Hikari Super camera (EDAX LLC, Mahwah, NJ, USA) at a step size of 2.5 µm, analyzed area 1076 µm × 1080 µm, acceleration voltage 30 kV, scanning speed 100 diffractograms per second, and 5 × 5 binning. EBSD maps were processed in 75× magnification. The data acquisition, analyses, and postprocessing were performed using the software TEAM 4.5 (EDAX LLC, Mahwah, NJ, USA) and EDAX OIM Analysis™ Version 8.0 (EDAX LLC, Mahwah, NJ, USA). EBSD and optical microscope images were analyzed using ImageJ version 1.52v software (Available online: https://doi.org/10.1038/nmeth.2089 (accessed on 25 July 2021)).

**Figure 4:**
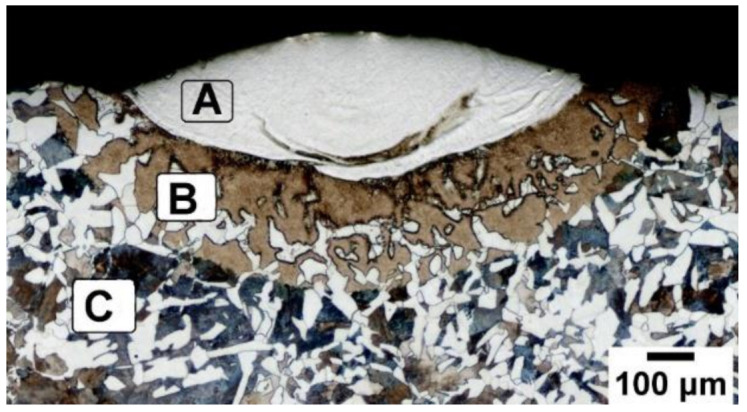
Transverse sectional optical image of the single-track.

A typical single-track transverse section from DED for the above process parameters is illustrated in Figure 4. The deposited material showed a distinct appearance compared to the base plate region. The important regions often used in this article are marked in Figure 4. An optical microscope image shows three regions. Region-A represents the melt pool solidified area. Region-B is the HAZ on the base plate. Region-C is the base plate material. A single-track deposition from DED is comparable to the usual welding process. Region-A is called the fusion zone (FZ). In this region, molten metal was created from powder and base plate material. The microstructure in this zone was the result of the material composition and solidification rate. Region-B is created due to thermal influence from the FZ. The HAZ is the region where the metallurgical process occurred in the solid-state. The HAZ is a complex region, and it is evident for several possible reactions such as grain growth, recrystallization, phase transformation, precipitate formation, and residual stress. These reactions are influenced by thermal history and material composition in this region. The solidification growth rate (R) defines the grain morphology. Dendrites morphology depends on the temperature gradient in the melt pool. The nucleation initiates at the solid-liquid interface, which forms a solid structure within the liquid phase. Further solidification depends on the latent heat of the metal from the solidification front. The heat from the solid-liquid interface dissipates to the base plate. The intensity of the heat dissipation to the base plate reflects in the HAZ by recrystallization and grain orientation.

The HAZ is an important region in the multilayer deposition process. During multi-layer deposition for solid structure construction, every beneath layer acts as a HAZ. Important factors to determine HAZ are thermal gradient, thermal diffusivity of the material, laser power, and scanning velocity [32]. In general, the HAZ size is a result of the interplay between process parameters and material properties.

### 2.3. Material

The solidification morphology and degree of epitaxy of microstructure growth of austenitic stainless steel 316L were relatively stable. Austenitic stainless steel 316L does not undergo phase transformation in the solid state. The change in microstructural morphology due to base plate preheating can be efficiently identified, and thus enabled us to understand the preheating influence on microstructure during the AM process. Therefore, austenitic stainless steel 316L was selected as the material for powder and base plate. The chemical compositions of the powder used for single track deposition and base plate material are listed in Table 2.

## 3. Results and Discussion

### 3.1. Microstructure Analysis

The microstructure developed from single track deposition on the base plate at room temperature is compared to the morphological change caused by different base plate preheating temperatures.

#### 3.1.1. Without Preheating (WPH)

**Figure 5:**
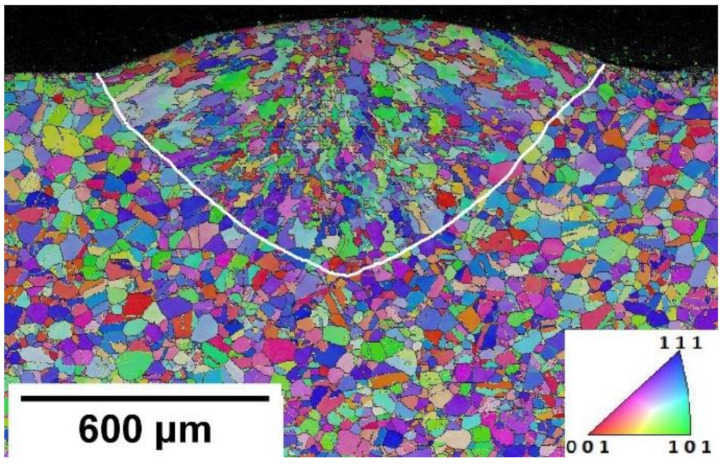
WPH condition EBSD map of Inverse Pole Figure (IPF) colored orientation image map with Pole Figure (PF) [001].

EBSD grain mapping shown in Figure 5 depicts grain formation in the FZ region. The white line is marked at the boundaries of columnar grains separating deformed grains from the base plate.

Fine grains were formed at the surface, which was directly exposed to the ambient temperature. The deposition chamber temperature was around 30 °C. The solidification in this region was mainly driven by the temperature gradient created due to convection heat flow from the melt pool surface to the deposition chamber. Heterogeneous nucleation could be induced by convection heat flow in the upper region and conduction in the lower portion of the melt pool to the base plate. The temperature gradient in the upper region of the melt pool could be due to forced convection. The shield gas was fed around the melt pool to protect it from oxidation. As the laser head was moving at the defined scanning speed, shielding gas was blown at the deposited material continuously as shown in Figure 2. This causes forced convection at the surface of the melt pool. This phenomenon was evident in all cases.

**Figure 6:**
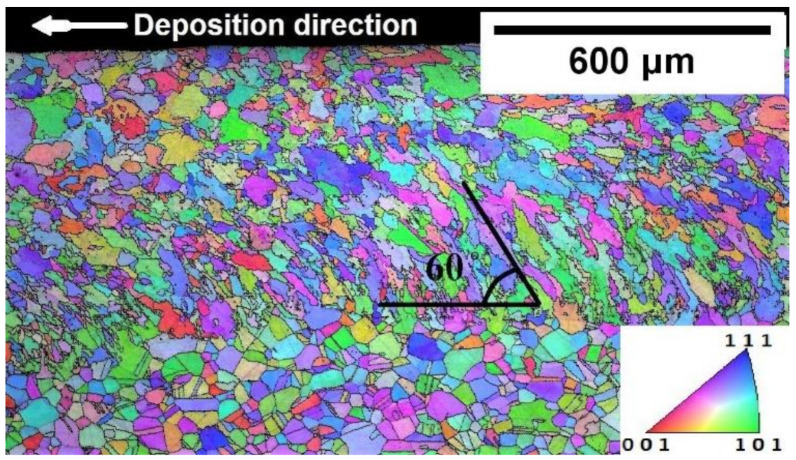
EBSD orientation map for longitudinal section deposited WPH with IPF [001].

The boundary formed between the melt pool and base plate in the bottom region was influenced by conduction heat flow. Grain nucleation and growth begin from the melt pool boundary progressing towards the center, known as directional solidification [33]. It is clearly evident from the transverse section image that the grain morphology in the top region is discontinued from the bottom solidification front, which is dominated by columnar grains. A similar grain morphology is reported for DED by other researchers [34,35]. The bottom region of the melt pool was in direct contact with the base plate, which was at room temperature. This led to a higher thermal gradient (G) in the liquid melt pool, whereas a lower thermal gradient was generated away from the base plate (around the centerline of the weld pool) [36]. Thus, coarse grains formation at the center of the weld line can be noticed in Figure 6.

Grains in the bottom region of the FZ are prone to incline towards deposition direction as shown in the EBSD longitudinal section image in Figure 6. It is well known that face centered cubic (FCC) crystals are most preferred to grow in <100> direction. Grains preferred to grow in <100> crystallographic direction from the base plate that is parallel to the local heat flow direction. Temperature gradient at the interface of the melt pool and base plate influence has a large impact on grain morphology. Therefore, grain morphology and temperature gradient could be controlled by the base plate preheating keeping all process parameters constant.

#### 3.1.2. Preheating Conditions

**Figure 7:**
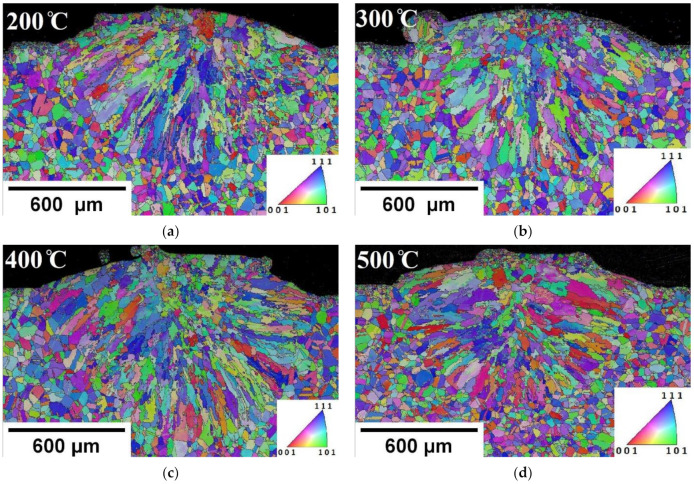

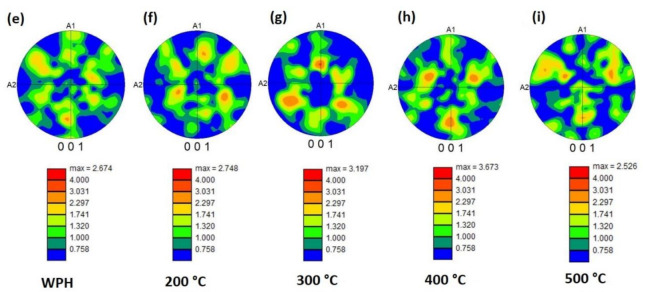
EBSD inverse pole figure maps for preheating base plate single track transverse section. (**a**) IPF—200 °C (**b**) IPF—300 °C (**c**) IPF—400 °C (**d**) IPF—500 °C and pole figures (**e**) WPH (**f**) 200 °C (**g**) 300 °C (**h**) 400 °C (**i**) 500 °C.

The EBSD analysis provided information on the development of the microstructure of individual base plate preheating temperatures. The color IPF maps represent random grain orientations along cross-sections for all states (perpendicular to laser track) in Figure 7. All images show the same trend of grain morphology similar to the WPH case presented in Figure 5. The bottom region of the FZ is dominated by columnar grains. In contrast, the topmost region of the FZ is dominated by fine grains. Grain growth and rearrangement, as observed on the IPF and PF shown in Figure 7b,g, respectively, proved that from base plate preheating temperature 300 °C there is an increase in the grain size fraction area with the increasing temperature.

The pole figures, which enable the representation of the preferred orientation (texture) in the analyzed material (Figure 7), proved that no strong texture was formed during single track deposition at a given base plate preheating temperature.

The elongated columnar grains perpendicular to the solidification front can be observed for all cases. This is dependent on the value of temperature gradient (G) and solidification rate (R) within the melt pool [33]. The melt pool creates a curved solid–liquid front for solidification, and the melt pool dimensions are dynamic. The G and R values change constantly from the base plate intersection to the centerline of the melt pool [37]. That creates competition for columnar grain growth. The columnar grain growth towards the centerline of the melt pool reflects the heat dissipation direction. The base plate preheating enlarges the depth of laser penetration. Interestingly, the preheating 500 °C condition had consistent columnar grain orientation and parabolic shape, shown in Figure 7d (parabolic shape can also be seen clearly in optical microscope image in Figure 10a). Solidification of the two solidification fronts from the sides of the melt pool boundary developed towards the centerline (vertical path of the laser penetration). This could be a result of the deeper laser penetration due to preheating, as remelting depth depends on the energy input [38].

**Figure 8:**
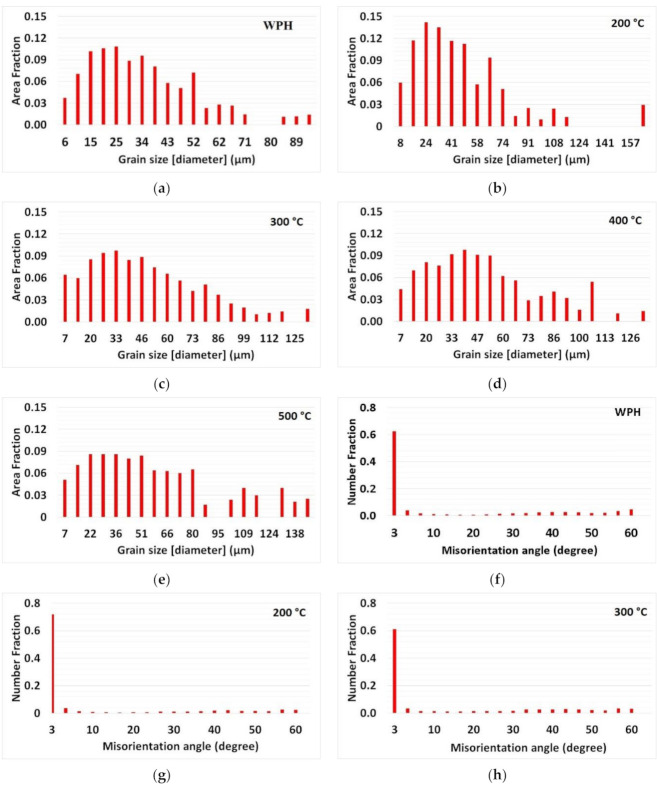

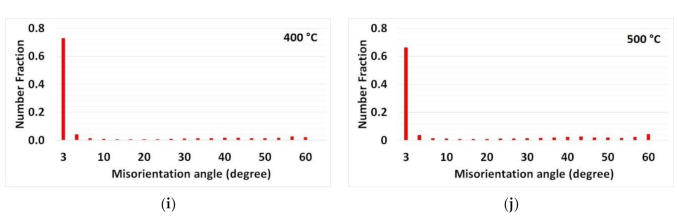
Grain size distribution data (**a**) WPH (**b**) 200 °C (**c**) 300 °C (**d**) 400 °C (**e**) 500 °C and grain boundary misorientation data (**f**) WPH (**g**) 200 °C (**h**) 300 °C (**i**) 400 °C (**j**) 500 °C.

It is important to notice that as the preheating temperature increases, the area fraction of coarse grains also increases, which is evident from the grains size calculation shown in Figure 8. The grain size distribution and misorientation angle were calculated, taking into account only the FZ regions (marked in Figure 5) from the respective IPF. Minimum and maximum grain size ranges lied between 6 μm and 94 μm for the WPH case, as depicted in Figure 8a. The base plate at room temperature acted as a sink, and it facilitated a high rate of heat flow from the melt pool. Interestingly, the grain size range did not vary much for 300 °C and 400 °C preheating conditions. The range fell largely between 7 μm and around 125 μm. The preheating at 200 °C resulted in a larger area fraction of the finer grains in the range between 16 μm and 66 μm. Based on the IPF and PF observation, the area fraction of large grains increased from preheating 300 °C, which can be noticed in the grain size distribution data illustrated in Figure 8c, whereas a significant amount of area occupied by large grains over 100 μm for preheating at 500 °C is depicted in Figure 8e. The larger grain size in the range from 131 μm to 145 μm with considerable area share can be noticed. This indicates that the preheating base plate has an influence on coarsening grains and extending area fraction for larger grains. A large area fraction of coarser grains was calculated for the 500 °C base plate preheating case. A large area fraction of coarse grains indicates the holding of heat due to preheating of the base plate. This could reduce the thermal mismatch between the melt pool generated from a high-energy concentric heat source and the base plate. Thus, preheating could minimize structural distortion while separating from the base plate. Low-angle grain boundaries (LAGB < 5°) occupied a large number fraction (between 0.6 and 0.73) for all cases. A relatively lower number fraction (maximum of 0.046) for high-angle grain boundaries (HAGB) is calculated for all cases. It is evident that preheating the base plate does not have an influence on grain boundary misorientation for single track deposition. Similar results are reported for 316L stainless steel fabricated via direct laser deposition (DLD), and an increase in the high-angle boundaries (60°) was observed for the heat-treated sample [39]. In contrast, SLM-processed Ti–45Al–2Cr–5Nb alloy preheating results in HAGB dominated microstructure [16].

**Figure 9:**
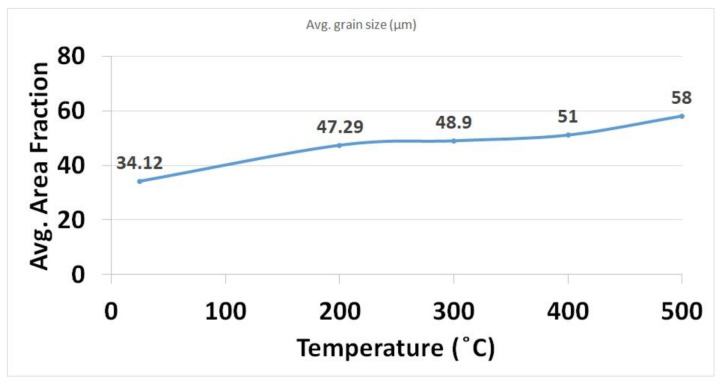
Average grain size plot for different base plate preheating temperature.

A shift in average area fraction can be noticed between WPH and 200 °C preheating, depicted in Figure 9. Further, for 300 °C and 400 °C preheating conditions, the average area fraction was nearly the same. However, a noticeable increase for 500 °C preheating conditions was observed. Preheating temperature has a negative impact on the cooling rate (T = G × R) [16]. Preheating temperature increased results in a reduced temperature gradient from the melt pool to the base plate. A negative impact on cooling rate during base plate preheating assisted in coarsening grains for large area fractions.

#### 3.1.3. Melt Pool Dimensions

**Figure 10:**
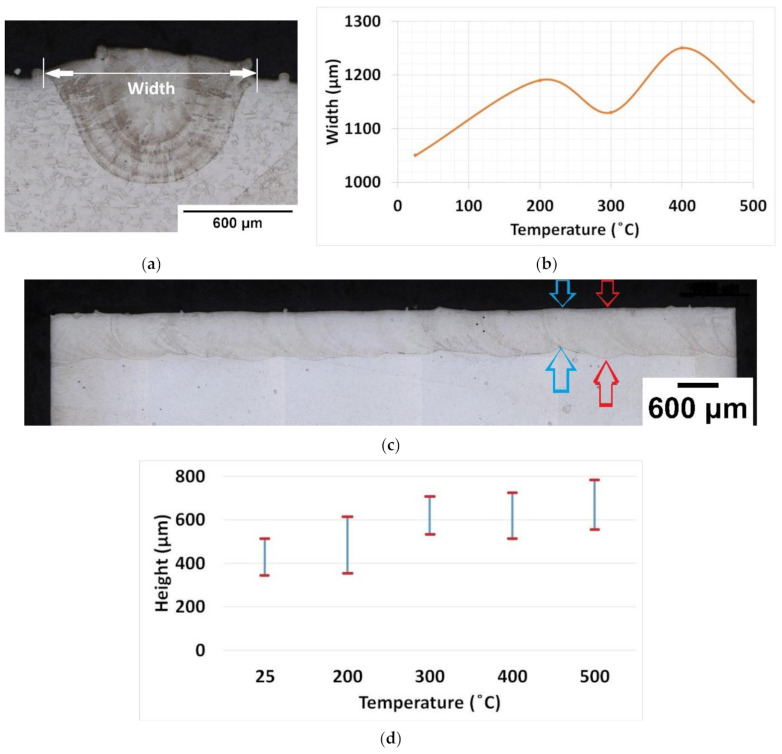
(**a**) Width measurement in the preheating 500 °C optical microscope image (**b**) Width plot for different base plate preheating temperature (**c**) Height measurement in the preheating 500 °C (**d**) Height measurement plot.

The width of the deposited material was measured using the optical microscope image, shown in Figure 10a, and height was measured for the longitudinal section, shown in Figure 10c.

The width of deposition almost remained constant for all cases, as shown in Figure 10b. A slight increase in width can be noticed for preheating temperature 400 °C. The results indicate a minor influence of preheating on the width of the deposit. The width of deposition is a major factor for welding. The width of the weld pool determines the strength of the joint, whereas in AM, multiple tracks are deposited with overlap on the subsequent track. The height of the melt pool and its consistency are important to determine the percentage of mixing materials and influence on the base plate material. The height of deposition was measured from the respective longitudinal section. The sections where minimum height measured was marked in blue indication and maximum height is shown in red color are presented in Figure 10c. Deposition height linearly increased as the preheating temperature increased. The base plate preheating enlarged the depth of laser penetration, which resulted in increasing the height of FZ. The height increased from preheating temperature 300 °C. More specifically, the largest height was reported for preheating 500 °C. The difference in minimum and maximum height reported from preheating temperatures 300 °C and above was nearly the same. Comparatively, the preheating 500 °C height of deposition appeared to be more uniform, as shown in Figure 10c.

#### 3.1.4. Longitudinal Section

The single track deposition was cut along the deposition direction, which provides a perpendicular view on a grain morphology to the deposition direction. The observations made in both transversal and longitudinal sections help to analyze grain formation in three dimensions.

**Figure 11:**
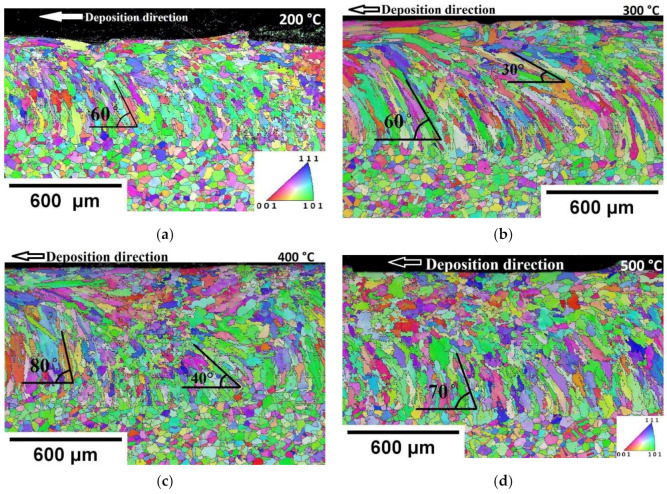
EBSD maps for preheating base plate single track longitudinal section. IPF colored orientation image map of preheating temperature (**a**) 200 °C (**b**) 300 °C (**c**) 400 °C (**d**) 500 °C.

The longitudinal view reveals grains that are inclined towards the deposition direction in the top region of the FZ. An enlarged grains growth, which was parallel to deposition direction, can be seen for preheating 300 °C and 400 °C. The preheating 500 °C longitudinal section was characterized by coarser equiaxed grains in the top region, as shown in Figure 11d.

The grains were oriented at an angle towards the deposition direction in the lower region of the FZ. It can be seen from Figure 11 that columnar grains were inclined towards the deposition direction in all cases. The grains were oriented around 60 ± 5° for WPH (Figure 6) and preheating 200 °C (Figure 11a). Typically, the grains orientate towards the moving melt pool in DED. The grain orientation around 60 ± 5° has been reported for unidirectional laser scanning in the multilayer deposition for DED [40,41,42].

It is worth noting that elongated columnar grains were developed for the preheating 300 °C and 400 °C, whereas 500 °C preheating assisted to develop a homogeneous microstructure. The longitudinal section of preheating 300 °C and 400 °C exhibited inhomogeneous columnar grains orientation. The grain orientation varied between 30° to 60° for preheating 300 °C and 40° to 80° for preheating 400 °C. Notably, at 500 °C preheating, the majority of columnar grains orientated at 70°.

## 4. Conclusions

Based on the results of microstructural analysis, it is evident that during single track deposition, the preheating of the base plate influences microstructure morphology. For preheating at 500 °C, an average area fraction of grain size distribution was almost doubled compared to the base plate without preheating. Base plate preheating for austenite 316L material single track deposition did not show any clear influence on the development of strong texture and a grain boundary misorientation angle.

The width of deposition was relatively stable for all cases. The height of deposition increased with the increase in preheating temperature. This indicates possible influence during a multilayer deposition where the previous layer acts as a deposition platform. The columnar grains orientation in the longitudinal section was observed to be inhomogeneous for base plate preheating conditions of 300 °C and 400 °C. At 500 °C preheating conditions, most of the columnar grains had an orientation angle around 70°. Thus, the preheating condition at 500 °C showed predominant effect on grain size and orientation. Varying the substrate preheating temperature proved the possibility to tailor the grains’ size and orientation. These results are crucial to understanding preheating effect on multi-layer deposition. Single-track deposition results can serve as a reference to measure the degree of influence during the base plate preheating at 500 °C for multilayer deposition. A significant transformation at 500 °C base plate preheating for single track deposition indicates the need to evaluate base plate preheating effect on microstructure, mechanical properties, and residual stress during multilayer solid structure deposition.

## Figures and Tables

**Table 1:** Process parameters for single track DED.

Process Parameter	Value
Laser power	500 W
Scanning speed	14 mm/s
Laser beam diameter	0.8 mm
Powder feed rate	3 g/min
Shielding gas and carrier gas	Argon
Shielding gas consumption	5 L/min
Laser standoff distance	9 mm

**Table 2:** Chemical composition (wt.%) of austenitic stainless steel 316L.

	Fe	Cr	Ni	Mo	Mn	Si
Powder	Bal.	17.2	10.4	2.3	1.3	0.8
Base Plate	Bal.	16.2	10.5	2.1	1.1	0.4

## Data Availability

Data available in a publicly accessible repository.
